# Obesity promotes immunotherapy efficacy by up-regulating the glycolytic-mediated histone lactacylation modification of CD8+ T cells

**DOI:** 10.3389/fphar.2025.1533464

**Published:** 2025-03-05

**Authors:** Kai-Xuan Wang, Dong-Min Shi, Xiao-Li Shi, Jing-Yuan Wang, Xing-Hao Ai

**Affiliations:** ^1^ Shanghai Lung Cancer Center, Shanghai Chest Hospital, School of Medicine, Shanghai Jiao Tong University, Shanghai, China; ^2^ Department of Medical Oncology, Changzheng Hospital, Naval Medical University, Shanghai, China; ^3^ Department of General Surgery, Nanjing Drum Tower Hospital, The Affiliated Hospital of Nanjing University Medical School, Nanjing, Jiangsu, China; ^4^ Department of Medical Oncology, Cancer Center, Zhongshan Hospital, Fudan University, Shanghai, China

**Keywords:** NSCLC, PD-1, immunotherapy, MCT1, histone lactacylation

## Abstract

The response rate of immune checkpoint blockade (ICB) therapy for non-small-cell lung cancer (NSCLC) remains limited. Recent evidence suggests that obese cancer patients are more likely to benefit from ICB therapy, however, the specific mechanism needs further research. In this study, we found that anti-PD-1 therapy was more effective in obese NSCLC patients compared to normal weight patients and this was verified in mouse NSCLC model. Further bioinformatics analysis indicated that the glycolytic metabolism was markedly elevated in obese NSCLC patients. *In vitro* co-culture experiment showed that both increased glycolysis of tumor cells and external addition of lactate promoted T cell PD-1 expression. And, PD-1 upregulation was related to monocarboxylate transporter 1 (MCT1)-mediated lactate transport and subsequent lysine lactylation of histones in T cells. Based on the aforementioned data, our study contributes to better application of anti-PD-1 therapy in NSCLC.

## Introduction

Lung cancer still poses a significant medical burden and economic loss worldwide (Li et al., 2023; Chen et al., 2022). Lung cancer consists of small-cell lung cancer (SCLC) and non-small-cell lung cancer (NSCLC) (Yang et al., 2019). NSCLC represents approximately 85% of all lung cancer cases and mainly consists of adenocarcinoma (LUAD), squamous cell carcinoma (LUSC), large cell carcinoma (LCC) (La Montagna et al., 2021), however, the 5-year survival rate of advanced NSCLC remains poor (around 20%) (Allemani et al., 2018).

Immune checkpoint blockade (ICB) is a powerful anti-cancer treatment modality for a wide variety of human malignancies (Page et al., 2014; Zeng et al., 2022). Blockade of PD-1 has achieved impressive clinical responses and revolutionized the treatment of many cancers including NSCLC (Peters et al., 2018). The 5-year survival rates of advanced NSCLC after anti-PD-1 immunotherapy Nivolumab or Pembrolizumab were 15.6% and 23.2%, respectively (Topalian et al., 2019; Garon et al., 2019). Nonetheless, the efficacy of ICB for NSCLC varies significantly among individuals, with an overall response rate of only 20%–30%. In fact, a large number of patients fail to respond to ICB or develop drug resistance (Lahiri et al., 2023). Therefore, how to elevate the efficacy of ICB in NSCLC still need further research.

Obesity is a global health problem and has been shown to be associated with the development of lung cancer (Blüher, 2019; Sung et al., 2019). Recent evidence suggests that obese cancer patients are more likely to benefit from ICB (You et al., 2021). Kichenadasse et al. (Kichenadasse et al., 2020) and Cortellini et al. (Cortellini et al., 2020) found that progression free survival (PFS)/overall survival (OS) in NSCLC patients with high body mass index (BMI) were longer than patients with low BMI during anti-PD-1/PD-L1 treatment. Similarly, obese patients in melanoma (Cortellini et al., 2019) and renal cell carcinoma (Sanchez et al., 2020) have been found to benefit more from immunotherapy. However, how obesity affects the ICB and how obesity affects the interaction between tumor cells and immune cells is still not clear.

In this study, we found that anti-PD-1 therapy was more effective in obese NSCLC patients from in-house data, and this was validated in mouse NSCLC model. Moreover, bioinformatics analysis indicated that the glycolytic metabolism was significantly upregulated in the obese NSCLC patients. *In vitro* co-culture experiment further showed that both increased glycolysis of tumor cells and external addition of lactate promoted T cell PD-1 expression. Further, PD-1 upregulation may be related to MCT1-mediated lactate transport and subsequent lysine lactylation of histones in T cells. Our study helps to reveal the mechanism by which obesity affects the efficacy of ICB.

## Materials and methods

### Reagents

Oxamic acid and Rotenone were from MCE. Glucose and lactate were from Solarbio.Anti-mouse PD-L1 monoclonal antibody and control IgG were purchased from Bio X Cell. High fat diet (45% fat, 60% fat) and control diet (10% fat) were from Medicience. FITC labeled anti-mouse CD45 mAb and PE labeled anti-mouse CD8a mAb were from Biolegend.

### Cell lines

The NCI-H23 human NSCLC cell line, LLC mouse lung cancer cells and Jurkat human T lymphocytic leukemia cells were from ATCC and maintained in DMEM or RPMI 1640 (HyClone) supplemented with 10% FBS (Bioexplorer), 100 U/mL penicillin (Yeasen), and 100 U/mL streptomycin (Yeasen) at 37°C in a humidified incubator containing 5% CO_2_.

### Western blot

Western blotting was performed as described previously (Feng et al., 2024). The primary antibodies were used as follows: PD-1 (Abcam), Histone H3, β-tublin (Cell Signaling Technology), pan-Kla (PTM BIO). Goat anti-rabbit IgG-horseradish peroxidase (HRP) (Proteintech) was the secondary antibody.

### qRT-PCR

qRT-PCR was performed as described previously (Feng et al., 2024). GAPDH was used as internal control for cells and tissues mRNAs assays. The primer sequences were used as follows.

**Table:** 

Primer	Sequence (5′-3′)
HK1(human)-F	CTGCGGTTGTGGATAAGA
HK1(human)-R	TGG​AGA​AGT​GTG​GAT​GAA​G
PDK1 (human)-F	AGATGAGTGACCGAGGAG
PDK1 (human)-R	CTT​GGA​AGT​ATT​GTG​CGT​AA
LDHA (human)-F	TGCCTGTATGGAGTGGAA
LDHA (human)-R	CCTGCTTGTGAACCTCTT
GAPDH (human)-F	GGA​GCG​AGA​TCC​CTC​CAA​AAT
GAPDH (human)-R	GGC​TGT​TGT​CAT​ACT​TCT​CAT​GG

### Lactate detection

The detection of lactate in cell line-cultured supernatants and single cell suspension from mouse tumor tissue samples was performed according to the manufacturer’s protocols of the lactate colorimetric test kit (Elabscience, E-BC-K002-M). For lactate detection in tissues, specifically, weighing 0.1 g of tissue was added in reagent one for adequate homogenization. The next step was centrifugation at 4°C and 12,000 g for 10 min to obtain the supernatant for the lactate test. The content of the detected lactate was shown as mmol/gprot.

### Mouse model

4-week female C57BL/6 mice were fed with control diet with 10% fat (CD10), high fat diet with 45% fat (HFD45) or 60% fat (HFD60) for 10 weeks to generate the normal weight mice (CD10 group) and high fat induced obese mice (HFD45 group and HFD60 group). The left lung of those mice was inoculated with cell suspension of LLC (CDX group) mice lung cancer cell (1 × 10^6^ cells) in a total volume of 50 μL (PBS: Matrigel = 4 : 1 as vehicle) or control vehicle (CTRL group) with insulin injection syringes to construct the orthotopic cell line derived xenograft model (Wang et al., 2020). CDX mice of CD10 and HFD60 groups were intraperitoneally injected anti-PD1 antibody (anti-PD1 group) or homologous antibody (Iso-IgG group) or solvent (Vehicle group) every 3 days from the 3^rd^ day after incubation of LLC mice cancer cell. The mice were intensively observed for survival status and tumor tissue were harvested when dying for Western blot, flow cytometry, and detection of intracellular lactate.

### Bioinformatics analysis

Tumor tissue bulk RNA-seq data from 15 patients of our previous NSCLC cohort (Tan et al., 2016) were analyzed by principal component analysis (PCA) and CIBERSORT (Newman et al., 2015) to compare the difference of immune cell infiltration between normal weight group and obese group. Gene set enrichment analysis (GSEA) and differentially expressed genes (DEGs) were also analyzed in RNA seq of our NSCLC cohort and TCGA colorectal adenocarcinoma cohort (because TCGA NSCLC cohort was without BMI) to characterize the biological process changes in obese group compared with normal weight group.

### Statistical analysis

All quantified data were expressed as the mean values ±standard error (mean ± SE). Student’s t-test for non-paired replicates was performed to identify statistically significant differences between treatment means. When p < 0.05 differences were considered significant. Progression free survival (PFS) and overall survival (OS) data were analyzed by Kaplan-Meier plot, and hazard ratio (HR) was estimated by Cox proportional hazards model with Logrank test.

## Results

### Immunotherapy is more effective in obese NSCLC patients

**FIGURE 1:**
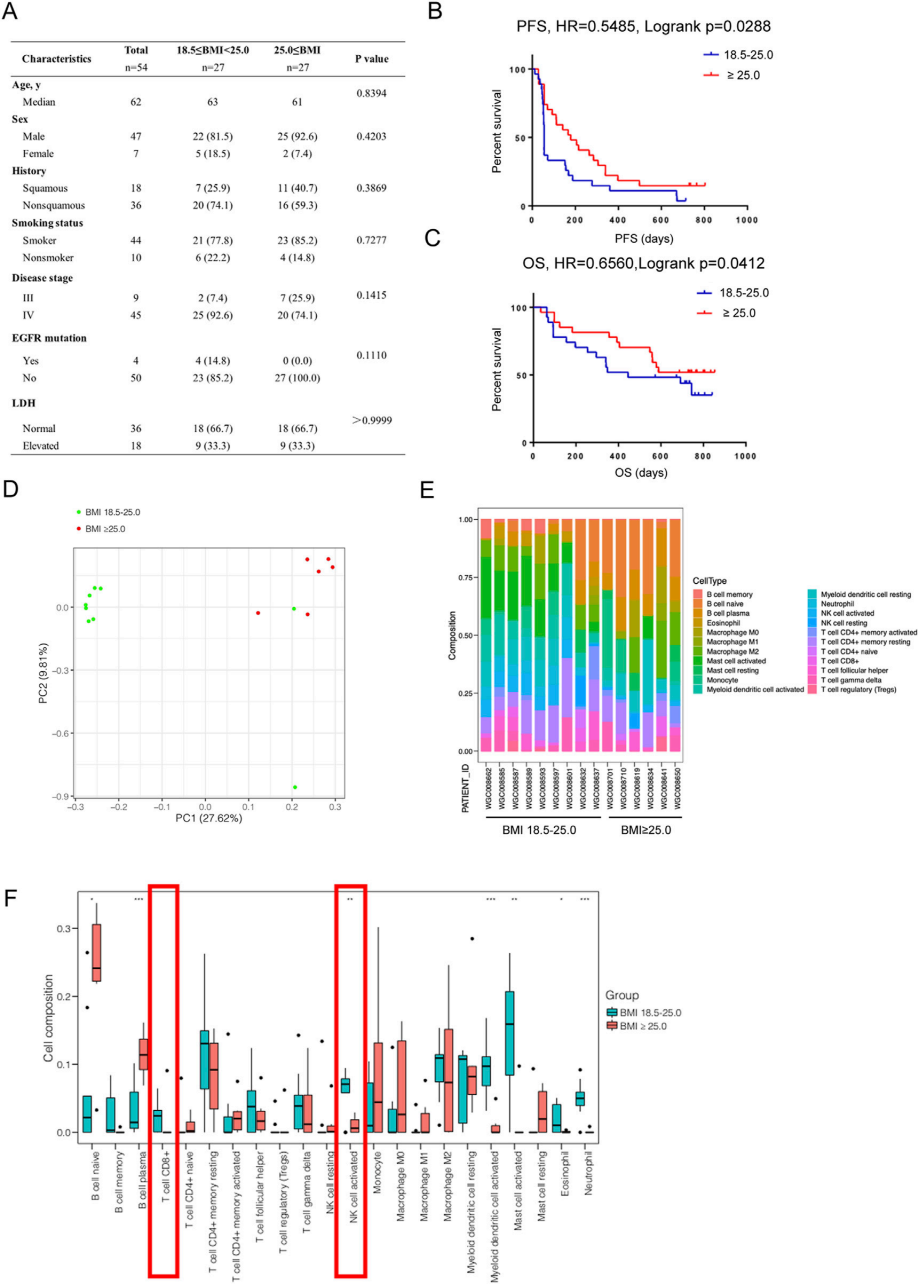
Immunotherapy is more effective in obese NSCLC patients. **(A)** Comparison of clinical characteristics between obese (BMI ≥ 25.0) and normal weight (18.5 ≤ BMI < 25.0) NSCLC patients from our center; **(B)** PFS and **(C)** OS comparison between obese and normal weight NSCLC patients treated by Nivolumab; **(D)** PCA analysis of transcriptome sequencing data of 15 NSCLC patients’ tumor tissues from our center showed BMI contributed the primary difference; **(E, F)** CIBERSORT analysis of tumor infiltrating immune cells and comparison between the normal weight group and obese group.

We examined the response of 54 NSCLC patients including obese group and normal weight group to Nivolumab in our center, patient information was showed in Figure 1A. There was no significant difference in clinic features between the normal weight group (18.5 kg/m^2^ ≤BMI <25.0 kg/m^2^) and the obese group (BMI ≥25.0 kg/m^2^), however, the obese group had longer mPFS (181.7 vs. 66.5 days, HR = 0.5485, Logrank p = 0.0288) and OS (NR vs. 452.5 days, HR = 0.6560, Logrank p = 0.0412) than the normal weight group (Figures 1B, C).

We then analyzed RNA-seq data from 15 NSCLC patients (6 obese and 10 normal) to obtain the percentage of various types of infiltrating immune cells in each tumor tissue sample (Figures 1D–F). The PCA result showed that the obese group and the normal weight group had significantly different clusters (Figure 1D). We found there was no difference in the number of CD8+ T cells between the normal weight group and obese group, suggesting that the differences in the function of CD8+ T cells should be further explored (Figures 1E, F). In addition, the activated NK cells in the obese group were significantly lower than those in the normal weight group, which was consistent with the findings of Bohn et al. in melanoma (Bohn et al., 2018).

### Anti-PD1 treatment is more effective in obese NSCLC mice

**FIGURE 2:**
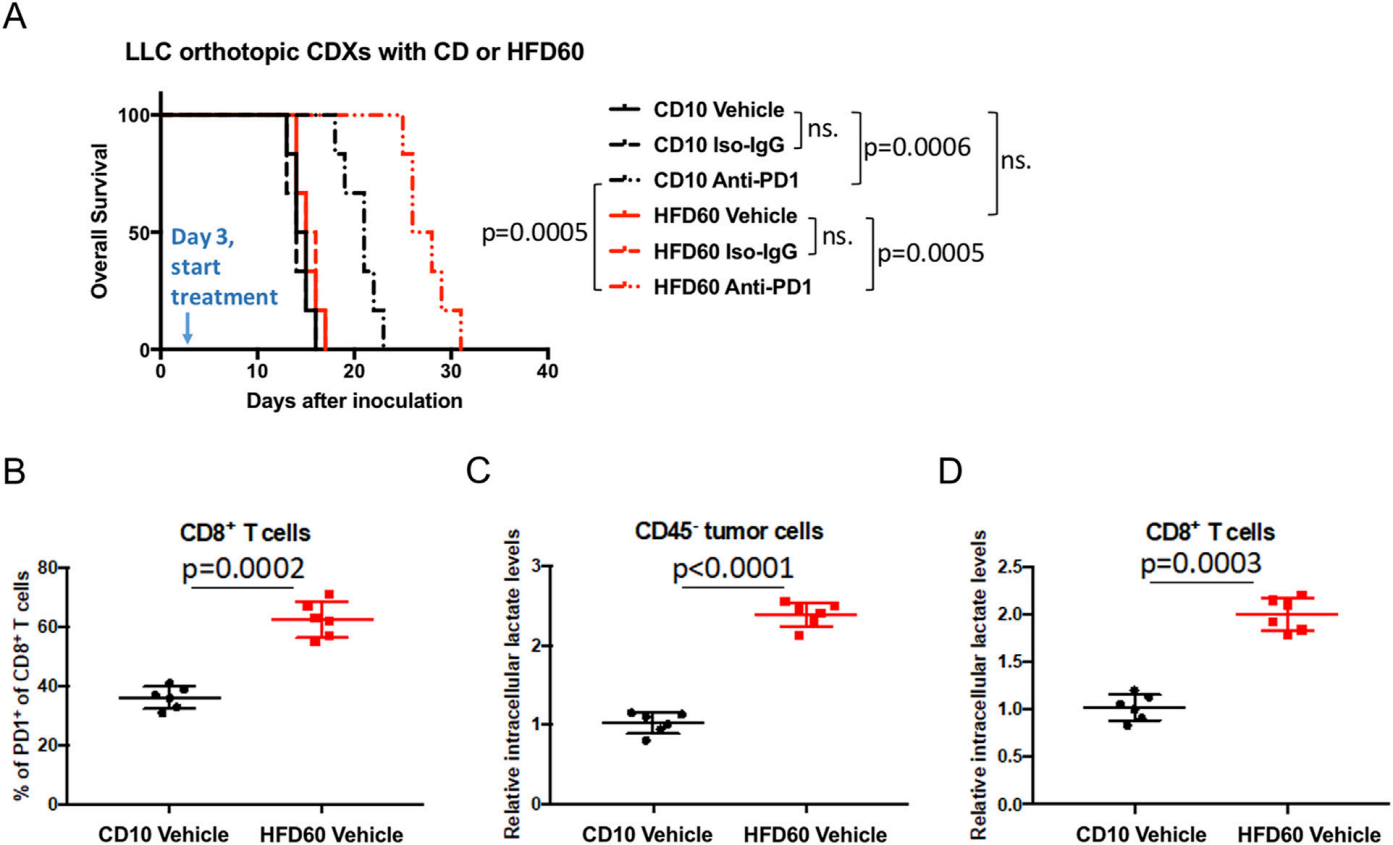
In orthotopic CDX mouse model of LLC cell line, the obese group showed better response to anti-PD1 treatment and higher intracellular lactate and PD1 expression on CD8+ T cells. **(A)** Normal weight (CD10) and obese (HFD60) mice were incubated with LLC cell line to generate orthotopic CDX model, and treated by anti-PD1, isotype IgG, and Vehicle. The overall survival was analyzed and compared among different groups; **(B)** PD-1 expression on CD8+ T cells by flow cytometry; Intracellular lactate analysis of **(C)** CD45^−^tumor cells and **(D)** CD8+ T cells.

We next investigated the response of the mice in the obese group and the normal weight group to anti-PD1 treatment. C57BL/6 normal weight mice (CD10 group, control diet with 10% fat) and high fat induced obese mice (HFD60 group, high fat diet with 60% fat) were inoculated with LLC lung cancer cells to construct the orthotopic cell line derived xenograft (CDX) model. Intraperitoneally injected anti-PD1 antibody (anti-PD1 group) or homologous antibody (Iso-IgG group) or solvent (Vehicle group) every 3 days from the 3rd day. The survival analysis in Figure 2A showed that there was no difference in OS between HFD60 Vehicle group and CD10 Vehicle group. And anti-PD1 antibody could prolong OS in both HFD60 Anti-PD1 group and CD10 Anti-PD1 group when compared with HFD60 Vehicle group and CD10 Vehicle group, while Iso-IgG and Vehicle had no effect on survival benefits. Moreover, HFD60 Anti-PD1 group showed extended OS when compared to CD10 Anti-PD1 group (median OS: 27 days vs 21 days). Then, CD45^−^tumor cells and CD8+ T cell in tumor tissues were sorted by flow cytometry, we found that tumor-infiltrating CD8+ T cells had higher PD1+ ratio (p = 0.0002) in HFD60 group than CD10 group (Figure 2B). Notably, CD45^−^tumor cells and tumor-infiltrating CD8+ T cells had higher intracellular lactate levels in HFD60 group than CD10 group (Figures 2C,D).

### Obese NSCLC patients have higher glycolytic metabolism than normal weight NSCLC patients

**FIGURE 3:**
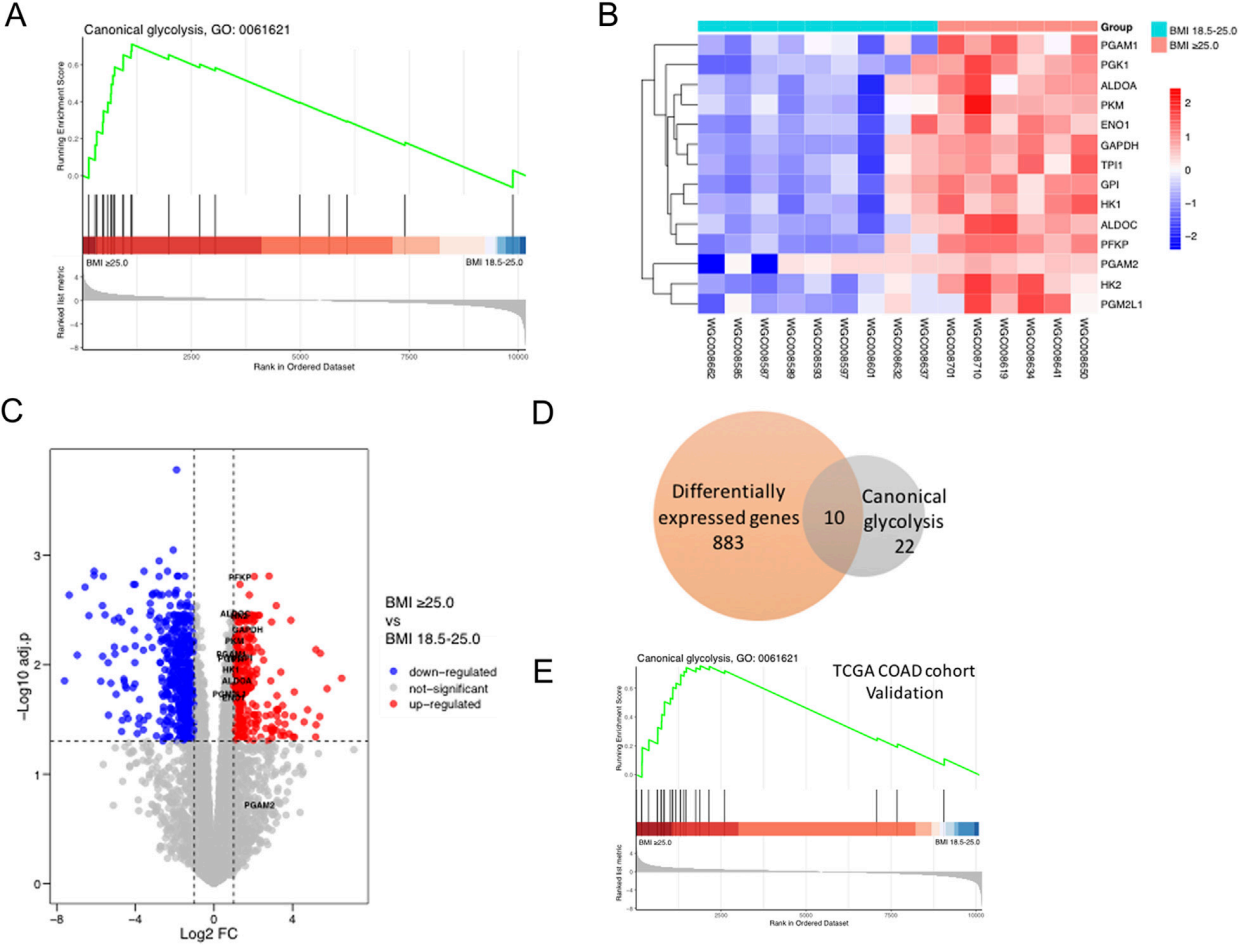
Obese NSCLC patients have higher glycolytic metabolism than normal weight NSCLC patients. **(A)** GO analysis of canonical glycolysis (GO 0061621) based on transcriptome data of 15 NSCLC patients’ tumor tissues from our center; **(B)** The heatmap of 14 main genes in canonical glycolytic pathway between the obese group and the normal weight group; **(C)** Volcano plot of DEGs between the obese group and the normal weight group; **(D)** Venn diagram of the intersection of DEGs and canonical glycolysis genes; **(E)** GO analysis of canonical glycolysis (GO 0061621) based on transcriptome data of colorectal adenocarcinoma patients in TCGA database.

GSEA analysis showed that canonical glycolysis signaling (GO: 0061621) was significantly upregulated in obese group compared to normal weight group (Figure 3A). And the expression levels of 14 glycolytic pathway genes (14/22, 64%) were upregulated in obese group compared to normal weight group (Figure 3B). Furthermore, 10 glycolytic pathway genes (10/22, 45%) were among those 883 differentially expressed genes (DEGs) between the normal weight group and the obese group (Figures 3C, D). In addition, we also found that canonical glycolysis signaling was obviously upregulated in obese group compared to normal weight group in the TCGA colorectal adenocarcinoma data (Figure 3E).

### Lactate released from tumor cells elevates T cell PD-1 expression

**FIGURE 4:**
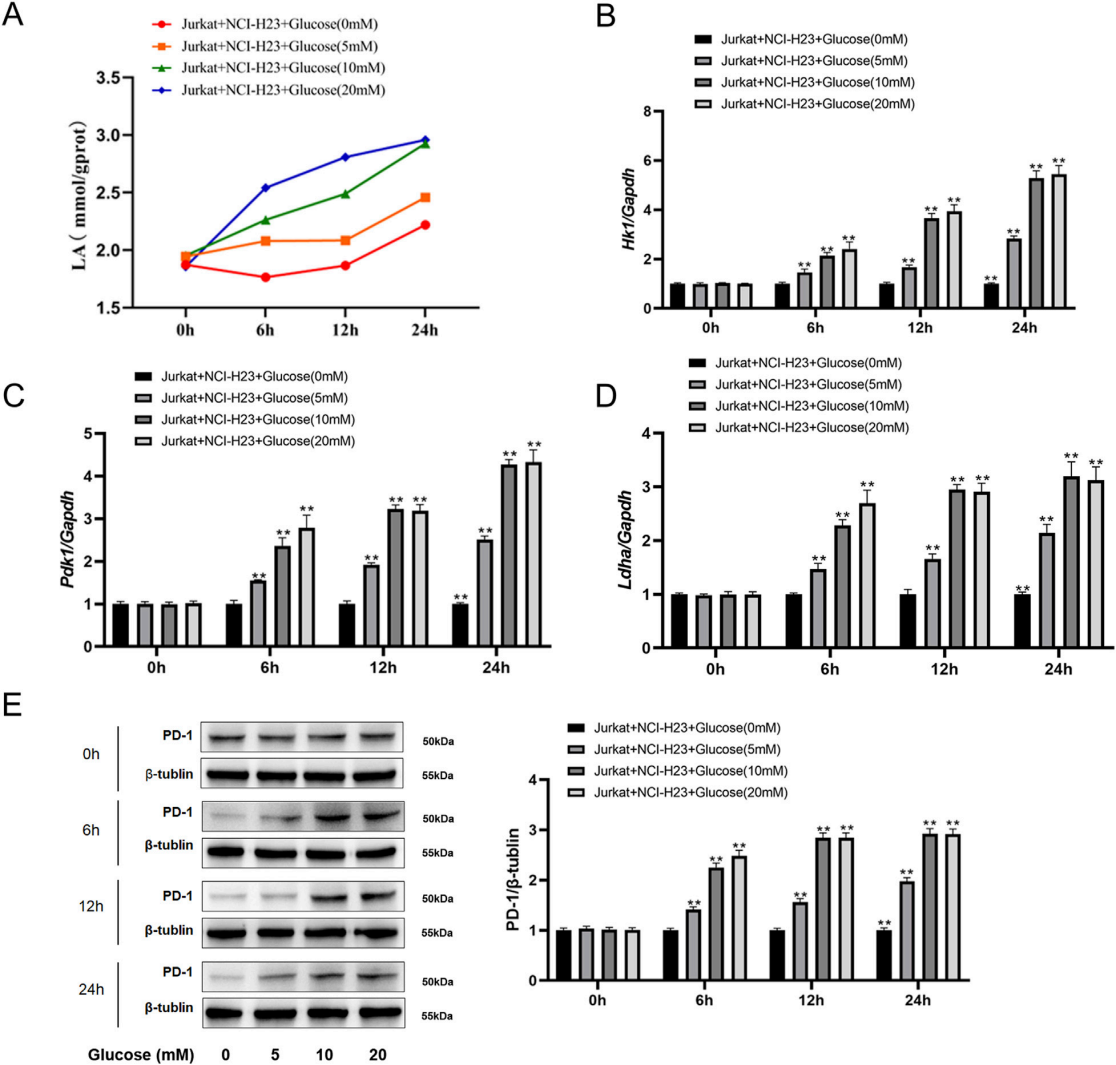
NSCLC tumor cells elevate PD-1 expression of T cells in a co-culture system. **(A)** Lactate levels in Jurkat cells when co-cultured with NCI-H23 cells at different concentrations of glucose in 0 h, 6 h, 12 h and 24 h; **(B)** HK1, **(C)** PDK1, **(D)** LDHA mRNA expressions by qPCR in NCI-H23 cells at different concentrations of glucose in 0 h, 6 h, 12 h and 24 h; **(E)** The protein levels of PD-1 in Jurkat cells when co-cultured with NCI-H23 cells at different concentrations of glucose in 0 h, 6 h, 12 h and 24 h.

Given that obese NSCLC patients have higher glycolytic metabolism than normal weight NSCLC patients (Figure 3) and tumor-infiltrating CD8+ T cells had higher intracellular lactate levels (Figures 2C, D), then we wanted to know if tumor cells secreted excessive lactate through glycolytic metabolism to promote T cell PD-1 upregulation. To unveil the effect of lung cancer cells on T cells, The NCI-H23 human NSCLC cells were co-cultured with Jurkat human T lymphocytic leukemia cells and treated with different concentrations of glucose. As shown in Figure 4A, lactate level in Jurkat cells increased with the increase of glucose concentration and accumulated over time. Meanwhile, the expressions of glycolytic metabolism related genes such as Hk1, Pdk1 and Ldha in NCI-H23 cells were increased with the growing concentration and treatment time of glucose (Figures 4B–D). In addition, the expression level of PD-1 in Jurkat cells in the co-culture system was also gradually upregulated with the treatment of glucose (Figures 4E, F).

**FIGURE 5:**
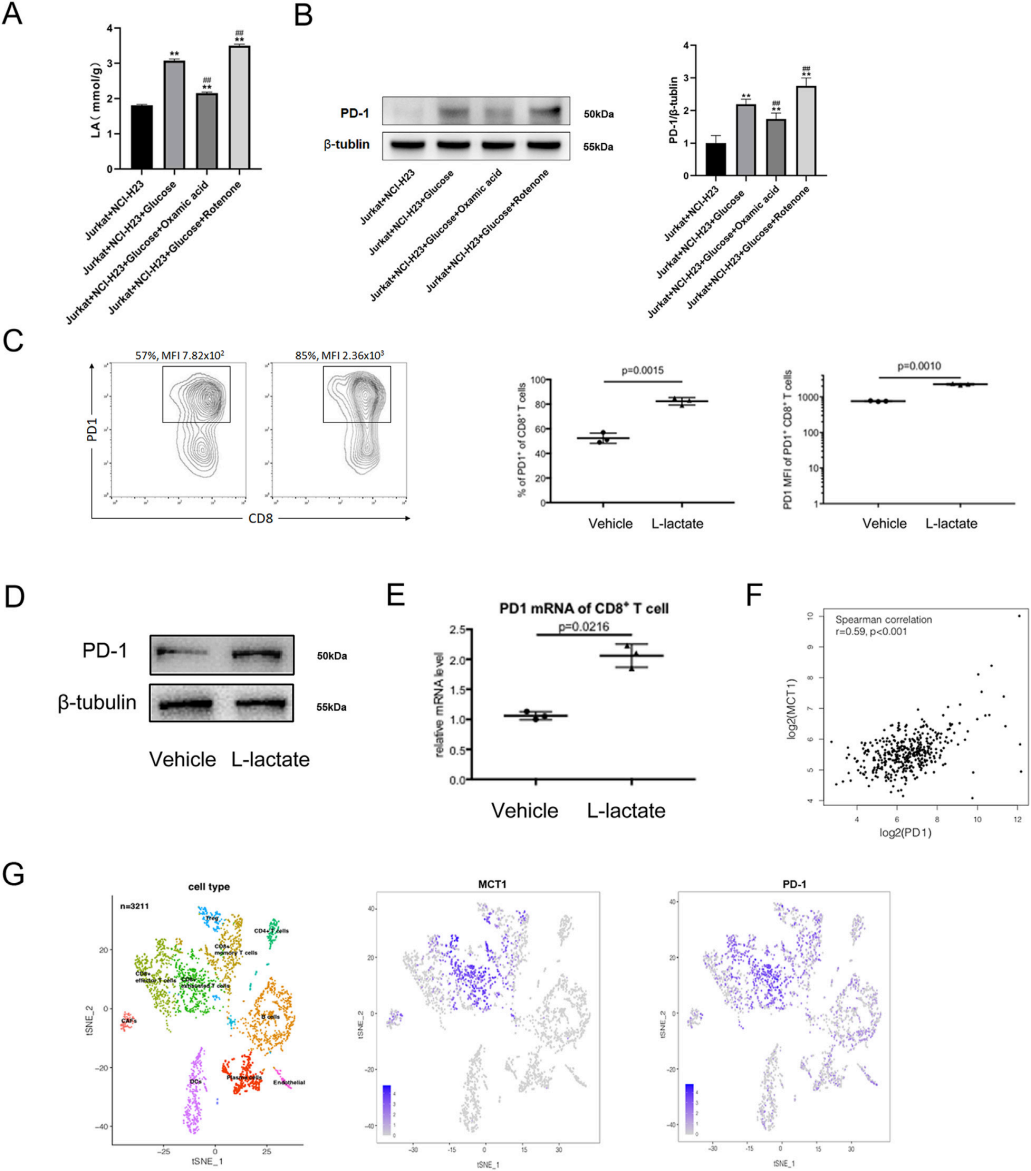
Lactate released from tumor cells elevates T cell PD-1 expression. **(A)** Lactate levels in Jurkat cells when co-cultured with NCI-H23 cells treated with glucose, glucose + oxamic acid, glucose + rotenone; **(B)** PD-1 protein expression in Jurkat cells when co-cultured with NCI-H23 cells treated with glucose, glucose + oxamic acid, glucose + rotenone by Western blot; Comparison of PD-1 expression on mouse spleen CD8+ T cells cultured with or without lactate by **(C)** flow cytometry, **(D)** Western blot, and **(E)** qPCR; **(F)** Correlation analysis of PD-1 and MCT1 in CD8+ T cells in tumor microenvironment by single-cell sequencing data from ArrayExpress database (data number. E-MTAB-6149); **(G)** The mRNA expressions of PD-1 and MCT1 in different clusters of tumor infiltrating immune cells by scRNA-seq.

To further explore the relationship between the lactate and PD-1, LDH-A inhibitor oxamic acid and mitochondrial respiratory chain inhibitor rotenone were employed. The results showed that oxamic acid decreased the lactate level of Jurkat cells in the co-culture system, as well as PD-1 expression, whereas rotenone caused the opposite results (Figures 5A, B). Above observations suggested that lactate from tumor cells might enhance the PD-1 expression in T cell.

To verify this result, we isolated CD8 + T cells from mouse spleens and treated cells with or without lactate. We found that lactate significantly upregulated the PD1+ ratio of CD8+ T cells (p = 0.0015) and PD1 fluorescence intensity (p = 0.001) (Figure 5C). Western blot and qPCR analysis demonstrated that lactate evidently elevated the protein and mRNA expression of PD-1 in T cells (Figures 5D, E). Since MCT1 is a well-known lactate transporter (Zhao et al., 2020), we next analyzed the expression of MCT1 in CD8+ T cells in tumor microenvironment by single-cell sequencing data from ArrayExpress database (data number. E-MTAB-6149). We found that the expression of MCT1 was significantly positively correlated with the expression of PD-1 (Figure 5F). In addition, PD-1 and MCT1 were highly expressed in CD8+ exhausted T cells in tumor-infiltrating immune cells (Figure 5G). Altogether, the upregulation of PD-1 in T cells may be caused by the secretion of lactate by tumor cells due to their active glycolytic metabolism.

### Lactate released from tumor cells elevates T cell PD-1 expression associated with the lysine lactylation of histones in T cells

**FIGURE 6:**
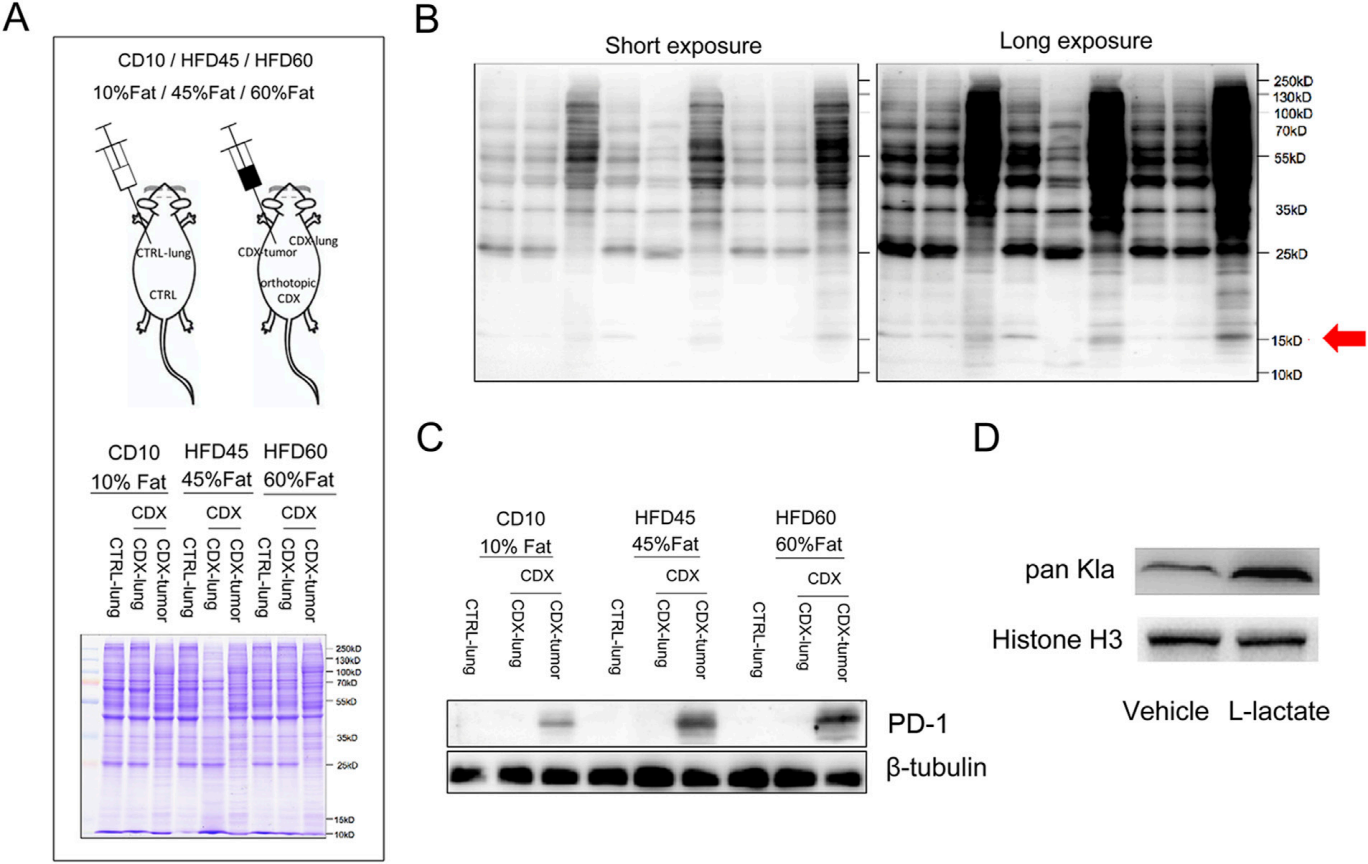
Lactate released from tumor cells elevates PD-1 expression on T cell associated with the lysine lactylation of histones in T cells. **(A)** Diagram of the experimental design: lung tissue of CTRL group (CTRL-Lung), CDX group (CDX-lung) and tumor tissue (CDX-tumor) from CDX models (CD10 group, HFD40 group, HFD60) were harvested 2 weeks later and lysine lactylation of protein were investigated by Western blot; **(B)** Lysine lactylation of protein in CTRL-Lung, CDX-lung, and CDX-tumor tissues of CD10 group, HFD40 group, and HFD60 group by Western blot; **(C)** The protein levels of PD-1 expression in CTRL-Lung, CDX-lung, and CDX-tumor tissues of CD10 group, HFD40 group, and HFD60 group by Western blot; **(D)** The histone protein lysine lactylation of mouse spleen CD8+ T cells treated with or without lactate by Western blot.

Meanwhile, the lung tissue of CTRL group (CTRL-Lung), CDX group (CDX-lung) and tumor tissue (CDX-tumor) from CDX models (CD10 group, HFD40 group, HFD60 group) were harvested 2 weeks later (Figure 6A) and lysine lactylation of protein were investigated by Western blot. As shown in Figure 6B, the lysine lactylation of protein in lung cancer tissues of normal weight or obese mice was significantly increased compared with normal lung tissue (including histones at 15 kDa), which was consistent with low glycolytic metabolism, low lactate level in normal lung tissues and high glycolytic metabolism, high lactate level in lung cancer tissues. Compared with normal weight mice, the lung cancer tissue of obese mice had higher lysine lactylation of protein, and the lysine lactylation of lung cancer tissue increased with the level of obesity (CD10 normal weight/HFD45 mild obesity/HFD60 severe obesity). We subsequently detected the expression of PD-1 protein and found that the expression of PD-1 in tumors of normal weight or obese mice was higher than that in lung tissues. Besides, the expression of PD-1 in tumors of obese mice was significantly higher than that of normal weight mice, and the expression of PD-1 also showed dose-dependent relationship with the level of obesity (Figure 6C). We then isolated CD8+ T cells from mouse spleens and treated CD8+ T cells with or without lactate and found that lactate obviously elevated the lysine lactylation of histones (Figure 6D). Collectively, the aforementioned results indicated that tumor cells upregulated the expression of PD-1 by releasing lactate to promote the lysine lactylation of histones in T cells.

## Discussion

The emergence of ICB has fundamentally changed the treatment of patients with advanced lung cancer (Sun et al., 2023). Compared with traditional chemotherapy, ICB causes fewer adverse events and significantly improves OS (Li et al., 2023). ICB alone or in combination with other treatments has brought hope for the treatment of lung cancer patients and is constantly developing (Konen et al., 2024). The FDA has approved a variety of immunotherapy drugs to treat NSCLC alone or in combination therapy. However, the different response of patients to ICB, as well as the intrinsic or acquired resistance of patients to ICB, are important questions in NSCLC research. There is no doubt that the application of personalized ICB to NSCLC patients is challenging.

The role of overweight or obesity in tumor is mainly reflected in two aspects: firstly, it promotes the occurrence and development of tumor; secondly, it affects the therapeutic effect of tumor (Saha et al., 2023). Obesity is associated with up to 49% of tumors, including a series of common cancers such as lung and colorectal cancer (Lauby-Secretan et al., 2016; Yu et al., 2018). Obesity inhibits chemotherapy efficacy by inducing inflammation and connective tissue hyperplasia in pancreatic cancer (Incio et al., 2016), enhancing MVP protein expression in breast cancer (Lehuédé et al., 2019), and inducing epithelial mesenchymal transformation in prostate cancer (Su et al., 2019). Obesity also inhibits the efficacy of anti-VEGF targeted therapy by enhancing the activity of FGF-2 pathway in breast cancer (Incio et al., 2018). Mechanically, previous studies focused on the effect of obesity on tumor microenvironment (TME) by inducing systemic inflammation, insulin resistance, sex hormone imbalance and other endocrine and metabolic disorders, as well as the upregulation of adipocytes and adipokines (Park et al., 2014; Renehan et al., 2015; Iyengar et al., 2016; Quail and Dannenberg, 2019). However, the mechanism of how obesity affects the metabolic interactions between tumor cells and immune cells, the tumor immune escape, and the impact on tumor immunotherapy is poorly understood. There is increasing clinical evidence that obese cancer patients are more likely to benefit from ICB and obesity is an independent predictor of better outcomes of immunotherapy PFS and OS, with a Hazard ratio (HR) of 0.71–0.76 compared to normal weight patients (You et al., 2021). More importantly, clinical data showed that obese cancer patients benefited more from ICB than normal weight in people with high PD-L1 expression (Kichenadasse et al., 2020; Cortellini et al., 2020). It is suggested that obesity may promote the efficacy of ICB due to affecting the interaction of tumor cell PD-L1 and immune cell PD-1. In this study, we found that anti-PD-1 therapy was more effective in obese NSCLC patients from our center, and this was validated in mouse NSCLC model (Figures 1, 2). Therefore, obesity may have an impact on the PD-L1/PD-1 pathway in the interaction between tumor cells and immune cells, and further exploration of its underlying mechanism will help reveal the therapeutic response mode and drug resistance mechanism of ICB.

Several studies have suggested the effects of obesity on T cells. Obese melanoma mice secrete leptin, and leptin activates the p-STAT3 pathway in peripheral blood CD8+ T cells through leptin receptor to bind to the PD-1 gene promoter region, up-regulating the transcription and expression of PD-1, thus inducing T cells of obese mice in a state of depletion with high expression of PD-1. In this case, obese melanoma mice had a better response to anti-PD-1 antibody, while there was no significant difference in the number of peripheral blood CD8+ T cells (Wang et al., 2019). However, the study did not solve the following problems: firstly, PD-1 expression was still increased after injection of leptin receptor knockout CD8+ T cells into obese mice, suggesting that in addition to leptin/pSTAT3 pathway activation, there are other pathways that upregulate the PD-1 expression of CD8+ T cells in obese mice. Secondly, the study only focused on peripheral blood CD8+ T cells, how TILs, as the main executive cell of TME, in obesity conditions remains to be studied. Here we found that glycolytic metabolism was significantly upregulated in the obese NSCLC patients (Figure 3). *In vitro* co-culture experiment further showed that the glycolysis level of tumor cells increased, and lactate released from tumor cells elevated T cell PD-1 expression (Figures 4, 5). Recent studies have shown that lactic acid produced by tumor cell glycolysis can be absorbed by Treg cells in TME via MCT1 and then promoting the inhibitory function of Treg cells (Watson et al., 2021). These results further support the effect of tumor cell glycolysis derived lactic acid on T cells.

In 2018, Zhao et al. reported that lactate upregulated the expression of homeostasis gene through histone lysine lactacylation (Kla), which transformed the M1 macrophages into M2 macrophages (Zhang et al., 2019). Meanwhile, homeostasis gene expression in tumor-associated macrophages was positively correlated with histone lysine lactacylation in mouse melanoma and lung cancer models. It is known that the transformation process of CD8+ effector T cell (Teff) to CD8+ exhausted T cell (Tex) is dependent on histone epigenetic modification (Khan et al., 2019; Beltra et al., 2020). It is not clear whether histone lysine lactacylation exists during the negative transformation of CD8+ effector T cells to CD8+ exhausted T cells. Our results suggest that there is a higher histone lysine lactylation in NSCLC tissues of obese mice, which is positively correlated with PD-1 expression (Figure 6).

In summary, obesity promote the expression of PD-1 by up-regulating the glycolytic-mediated histone lactacylation modification of CD8+ T cells in the TME, thus affecting the efficacy of ICB. Our findings provide new insights for better application of PD-1/PD-L1 therapy in NSCLC.

## Data Availability

The original contributions presented in the study are included in the article/supplementary material, further inquiries can be directed to the corresponding author.

## Abbreviations

ICB, immune checkpoint blockade; NSCLC, non-small-cell lung cancer; SCLC, small-cell lung cancer; LUAD, lung adenocarcinoma; LUSC; Lung squamous cell carcinoma; LCC, large cell carcinoma; HR, hazard ratio; GSEA, Gene set enrichment analysis; DEGs, differentially expressed genes; PFS, progression free survival; OS, overall survival; CDX, cell line derived xenograft; BMI, body mass index; PCA, principal component analysis; MCT1, Monocarboxylate transporter 1.
